# Supplementary material for: A novel method for the capture-based purification of whole viral native RNA genomes
Source: AMB Express. 2019 Apr 8;9:45. doi: 10.1186/s13568-019-0772-y (PMC6453989; doi:10.1186/s13568-019-0772-y)

**Additional Materials for:**

**A Novel Method for the Capture-based Purification of Whole Viral Native RNA Genomes**

Cedric Chih Shen Tan^1,2,3†^, Sebastian Maurer-Stroh^4^, Yue Wan^1^, October Michael Sessions^5^, Paola Florez de Sessions^1^

^1^ Genome Institute of Singapore (GIS), Agency for Science, Technology and Research (A*STAR), Singapore

^2^A*STAR Graduate Academy, Singapore

^3^University College London, United Kingdom

^4^Bioinfomatics Institute (BII), A*STAR, Singapore

^5^National University of Singapore, Saw Swee Hock School of Public Health, Singapore

^†^Correspondence to: Cedric CS Tan ([cedriccstan@gmail.com](mailto:cedriccstan@gmail.com))

| Table S1: Reagents and volumes for hybridization step. | | |
| --- | --- | --- |
| **Components** | **Volume (uL)** |  |
| 2x Hybridization Buffer | 30 |  |
| Hybridization Component A | 12 |  |
| Diluted Baits | 4 |  |
| Nuclease-free water | 10 |  |

Table S2: Reagents and volumes for RT-qPCR.

| **Components** | **Volume (uL)** |
| --- | --- |
| RNA in Nuclease-free Water | 1.0 |
| 2x QuantiTect Probe RT-QPCR Master Mix Buffer | 5.0 |
| QuantiTect RT Mix | 0.1 |
| Nuclease-free Water | 1.65 |
| 10 µM Forward Primer | 0.9 |
| 10 µM Reverse Primer | 0.9 |
| 10 µM Probe | 0.45 |
| Total Volume | 10 |
|  |  |

Table S3: RT-qPCR program set on Applied Biosystems ViiA 7 Real Time-PCR System.

| **Sequence Name** | **Sequence (5’-3’)** |
| --- | --- |
| DENV1 F | ACTAGYGGTTAGAGGAGACC |
| DENV1 R | GGTCTCCWCTAACCTCTAGT |
| DENV1 Probe | FAM-ACCAGGGRAAGCTGTAYCYT-BHQ |
| DENV1 Standard | ACTAGTGGTTAGAGGAGACCCCTCCCGAAACACAACGCAGCAGCGGGGCCCAACACCAGGGGAAGCTGTACCCTGGTGGTAAGGACTAGAGGTTAGAGGAGACC |
| GAPDH F | GATTCCACCCATGGCAAATTC |
| GAPDH R | ATTTCCATTGATGACAAGC |
| GAPDH Probe | FAM-CGTTCTCAGCCTTGACGGTGCCA-BHQ |
| GAPDH Standard | TATGATTCCACCCATGGCAAATTCCATGGCACCGTCAAGGCTGAGAACGGGAAGCTTGTCATCAATGGAAATCCCATCA |

Table S4: Primers, probes and standards used with the respective modifications for RT-qPCR.

| **Program name** | **Temperature/°C** | **Time/min** | **Number of Cycles** |
| --- | --- | --- | --- |
| Reverse Transcription | 48 | 30 | 1 |
| PCR Initial Activation Step | 95 | 15 |  |
| Denaturation | 95 | 0.25 | 40 |
| Combined Annealing/Extension | 55 | 1 |  |
|  | | | |

Table S5: Reagents and volumes used for polyadenylation of RNA samples.

| **Components** | **Volume (uL)** |
| --- | --- |
| **RNA** | 24 |
| **10x E. Coli Poly(A) Polymerase Reaction Buffer** | 3.2 |
| **ATP (10mM)** | 3.2 |
| **E. Coli Poly(A) Polymerase** | 1.6 |

Figure S1: DENV1 and GAPDH standard curves used for calculation of primer efficiency.

Figure S2: Coverage plot against nucleotide position on DENV1 reference for pre-capture MinION sequencing run.


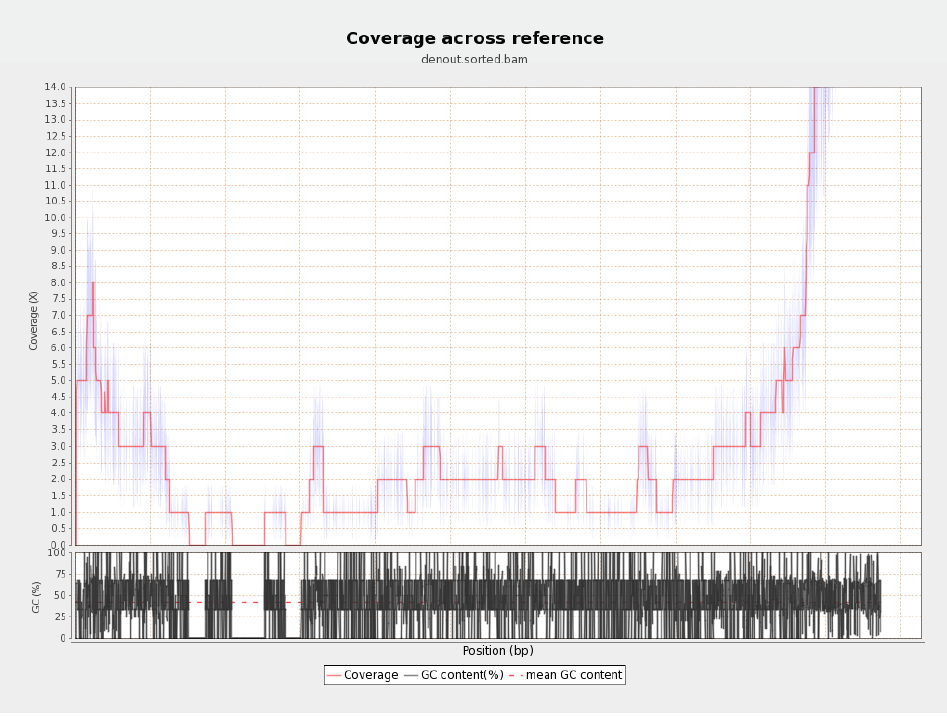


Figure S3: Coverage plot against nucleotide position on DENV1 reference for post-capture MinION sequencing run.


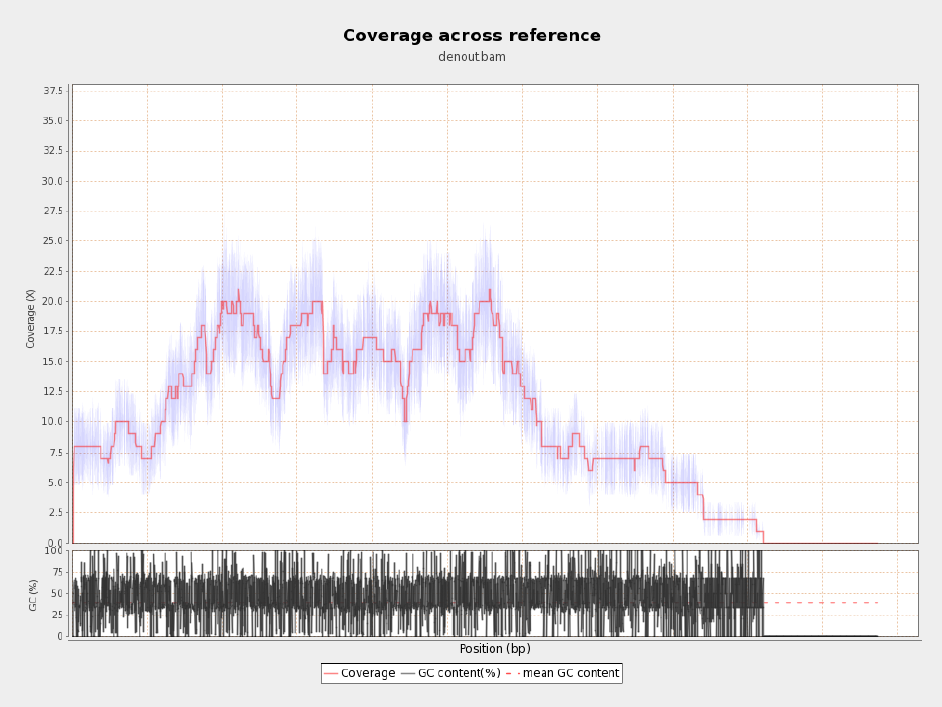


Figure S4: Coverage plot against nucleotide position on DENV1 reference for concentrated post-capture group MinION sequencing run.


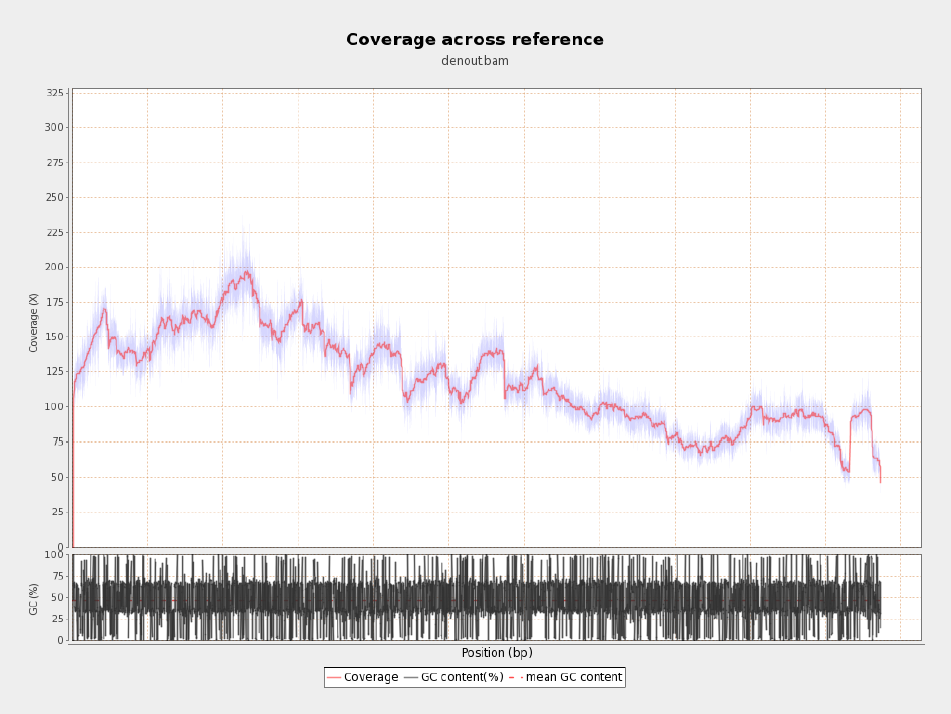

Supplement: Supplementary file 1 — Additional file 1: Table S1. Reagents and volumes for hybridization step. Table S2. Reagents and volumes for RT-qPCR. Table S3. RT-qPCR program set on Applied Biosystems ViiA 7 Real Time-PCR System. Table S4. Primers, probes and standards used with the respective modifications for RT-qPCR. Table S5. Reagents and volumes used for polyadenylation of RNA samples. Figure S1. DENV1 and GAPDH standard curves used for calculation of primer efficiency. Figure S2. Coverage plot against nucleotide position on DENV1 reference for pre-capture MinION sequencing run. Figure S3. Coverage plot against nucleotide position on DENV1 reference for post-capture MinION sequencing run. Figure S4. Coverage plot against nucleotide position on DENV1 reference for concentrated post-capture MinION sequencing run. [file 13568_2019_772_MOESM1_ESM.docx]
